# The AerVia supraglottic airway device versus the endotracheal tube in neonatal delivery room resuscitation: a prospective cohort study

**DOI:** 10.3389/fped.2026.1927663

**Published:** 2026-09-17

**Authors:** Naser Aldain A. Abu Lehyah, Alaa M. Abusabha, Abeer A. Hasan, Rahaf A. Al-Qaryouti, Qasem A. Shersheer, James J. Cummings, Scott O. Guthrie

**Affiliations:** 1 Division of Neonatology, Department of Pediatrics, Women’s and Children’s Hospital, Al Bashir Hospitals, Amman, Jordan; 2 Department of Pediatrics, Women’s and Children’s Hospital, Al Bashir Hospitals, Amman, Jordan; 3 Division of Neonatology, Department of Pediatrics, Albany Medical Center, Albany, NY, United States; 4 Division of Neonatology, Department of Pediatrics, University of Tennessee College of Medicine, Chattanooga, TN, United States

**Keywords:** AerVia, endotracheal intubation, laryngeal mask airway, neonatal resuscitation, preterm infant, supraglottic airway device

## Abstract

**Objective:**

To evaluate a supraglottic airway device (SAD) as an alternative to the endotracheal tube (ETT) in infants requiring resuscitation in the delivery room.

**Study design:**

Newborns requiring resuscitation beyond face mask ventilation due to inadequate ventilation or persistent bradycardia at Al Bashir Hospital in Amman, Jordan, were resuscitated using the AerVia SAD (*n* = 73) and compared with a historic cohort resuscitated by the ETT (*n* = 80).

**Results:**

AerVia and ETT cohorts had similar mean gestational age (32.0 ± 2.8 vs. 32.7 ± 2.3 weeks, *p* = 0.13) and birth weight (1.63 ± 0.44 vs. 1.68 ± 0.37 kg, *p* = 0.48). Although several comparisons demonstrated clinically important differences favoring the AerVia SAD, not all reached conventional statistical significance. First-attempt success was higher for AerVia than for the ETT (99% vs. 90%, *p* = 0.055). AerVia placement required fewer attempts (1.0 ± 0.1 vs. 1.1 ± 0.3, *p* = 0.07), was faster (9 ± 2 vs. 11 ± 3 s, *p* = 0.001), and resulted in comparable return of spontaneous breathing (45 ± 12 vs. 42 ± 8 s, *p* = 0.07). In a multivariate analysis, use of an SAD was associated with increased odds of first-attempt success compared with the ETT (adjusted odds ratio 9.28, 95% CI 0.89–96.76, *p* = 0.063). Complication rates were comparable for AerVia (18% vs. 23%, *p* = 0.55); the most common complication in the AerVia cohort was mild abdominal distension (*N* = 6) and in the ETT cohort airway trauma (*N* = 12). Eighty-three infants were <34 weeks’ gestation (AerVia = 47, ETT = 36), and comparisons in this subgroup were similar.

**Conclusion:**

The AerVia SAD was associated with less airway trauma compared with the ETT. Although placement appeared faster and required fewer attempts, these differences did not reach statistical significance. SADs appear to be a reasonable alternative to an ETT for advanced neonatal resuscitation in the delivery room. Our findings suggest that appropriately sized neonatal SADs may also be feasible in selected infants born <34 weeks’ gestation and support further studies.

## Highlights

- The AerVia supraglottic airway device (SAD) achieves faster, higher first-attempt success compared with the endotracheal tube (ETT) in resuscitation
- Severe complications are fewer with the AerVia SAD than with the ETT
- There is an evidence gap when it comes to SADs in infants born <34 weeks’ gestation; our research addresses this gap and supports its use for resuscitation in such infants

## Introduction

Positive-pressure ventilation (PPV) is the cornerstone of neonatal resuscitation, yet establishing a secure airway remains technically challenging. This is particularly true in preterm and very-low-birth-weight (VLBW) infants (1). Face mask ventilation (FMV), the initial standard method for delivering PPV, can be ineffective because of mask leak and airway obstruction, with failure rates of approximately 19% in randomized trials (2, 3). Once FMV fails, endotracheal intubation (ETT) has historically been the standard advanced airway intervention. This, however, requires advanced laryngoscopy skills, which have become increasingly rare among trainees and frontline providers (4). As an invasive procedure, laryngoscopy is associated with well-documented risks of airway trauma, oxygen desaturations, and bradycardia (5). Preterm neonates are especially vulnerable because not only is the neonatal airway smaller, but also there are anatomical and tissue differences that make laryngoscopy technically challenging to perform in a smaller infant (6).

Supraglottic airway devices (SADs) offer a less invasive alternative that can be placed rapidly without laryngoscopy. One of the earliest prospective evaluations of using the device in the delivery room demonstrated the feasibility and speed of the technique in 21 term and late-preterm neonates (≥35 weeks’ gestation, ≥2.5 kg) requiring PPV at birth. An SAD was successfully inserted on the first attempt in 100% of cases, with a mean insertion time of 8.6 ± 1.4 s (range 7–12 s) (7). Systematic reviews and meta-analyses have since confirmed that SADs are superior to FMV in late-preterm and term infants, reducing the need for ETT placement and shortening time to stabilization (2, 8, 9).

The 2025 American Heart Association (AHA) Neonatal Resuscitation Guidelines and the 2025 International Liaison Committee on Resuscitation (ILCOR) Consensus on Science with Treatment Recommendations now endorse SADs as a reasonable alternative to the ETT if FMV proves unsuccessful in infants ≥34 weeks’ gestation and support their use as the primary interface for PPV instead of FMV in this population (10, 11). Both guidelines, however, explicitly note the paucity of data for infants <34 weeks’ gestation. This leaves a critical evidence gap for the most vulnerable neonates who most frequently require advanced airway support (12). Recent device innovations have produced size-specific neonatal SADs (13), which allowed us to address the evidence gap regarding SADs in a prospective cohort that included many preterm infants.

## Methods

### Study group

This prospective observational study was conducted at the Women's and Children's Hospital of Al Bashir Hospital in Amman, Jordan. This is the country's largest governmental tertiary referral center, serving a high-volume, high-risk obstetric population with approximately 450 emergency deliveries per month. The facility includes a 400-bed hospital with an average of approximately 14,000 annual births and a 90-bed Level III neonatal intensive care unit (NICU) capable of providing comprehensive neonatal care. Since 2019, the study team has routinely incorporated SADs for surfactant administration through laryngeal or supraglottic airways (SALSA) (14) and has progressively expanded SAD use for delivery-room resuscitation.

### Study device

The AerVia neonatal supraglottic airway (ONY Biotech, Amherst, NY, USA) was used as the study device. AerVia is a purpose-built supraglottic airway specifically designed for neonatal anatomy. It is available in four sizes accommodating birth weights from <1,000 to 5,000 g. The device features a non-inflatable cuff with airway sealing ridges and an epiglottic holding pocket. It was inserted according to the manufacturer's instructions for use to establish and maintain a patent airway during PPV in the delivery room (15).

### Identification of patients for treatment

All newborns born between 1 January and 31 August 2025 and who required delivery-room resuscitation beyond FMV were eligible for treatment. A prospective cohort of infants managed with the AerVia SAD was compared with a historical cohort of infants resuscitated with ETT placement. The historical ETT cohort was drawn from patients managed with conventional intubation at the same institution during the period surrounding the introduction of SAD techniques in 2019, and had similar data collected prospectively, as previously described (14).

### Delivery room care

Neonatal resuscitation followed Neonatal Resuscitation Program (NRP) guidelines and the providers using AerVia received device-specific training and hands-on familiarization before clinical implementation. When FMV failed to achieve effective ventilation as determined by inadequate chest rise and/or persistently low or declining heart rate despite corrective steps, the clinical team then proceeded to advanced airway placement. The duration of FMV prior to escalation was guided by real-time physiologic assessment.

There were no predefined criteria for choosing AerVia versus an ETT. The choice of airway device was determined by the attending neonatologist and resuscitation team based on real-time clinical assessment, including the infant's clinical condition, urgency of establishing effective ventilation, anticipated airway difficulty, provider familiarity with the device, and device availability. Infants requiring immediate airway control or those perceived to be more critically ill were more likely to undergo endotracheal intubation according to standard clinical practice. In the absence of a formal allocation protocol and the difficulty of doing so for resuscitation studies, this resulted in a pragmatic, observational comparison.

Airway trauma was assessed clinically during and immediately after airway placement by the resuscitation team and prospectively documented using a standardized case-report form. Airway trauma was defined as clinically apparent injury related to airway instrumentation, including visible blood on the device after removal, oral or pharyngeal mucosal injury, or other signs of traumatic airway manipulation.

### Data collection

All data were collected prospectively by trained research personnel using a standardized case-report form. For each infant, demographic and perinatal variables were recorded, including gestational age; birth weight; mode of delivery; gender; singleton versus multiple gestation; and Apgar score at 1 min of age.

Procedural variables included the number of airway placement attempts; time to successful airway insertion; and time to return of spontaneous respiratory effort. Clinical outcome measures included Apgar scores at 5 and 10 min of age. Performance and safety endpoints included first-attempt success rate; incidence of airway trauma; incidence of pneumothorax; and overall complication rate.

Procedural times were prospectively recorded by trained research personnel independent of the resuscitation and airway placement teams. The observers were not involved in clinical decision-making or airway procedures. Time to successful placement was measured from device insertion until effective ventilation was confirmed, and time to return of spontaneous respiration was measured from successful placement until sustained spontaneous breathing was observed.

A subgroup analysis was performed for infants born <34 weeks’ gestation.

The study protocol was reviewed and approved by the Institutional Review Board of the Jordanian Ministry of Health (IRB No. MOH/REC/2025/417). In accordance with institutional policy, all pregnant women provide written informed consent upon admission, with two copies obtained—one for maternal management and a second designated for the newborn—to be used if NICU admission is required, thereby avoiding delays in time-critical situations. This consent includes approval for maternal management and newborn care.

Data analysis was conducted by the authors.

### Statistical analysis

Continuous variables are presented as mean ± standard deviation and compared using the independent samples t-test. Ordinal variables are reported as median with interquartile range (IQR) and compared using the Mann–Whitney U test. Categorical variables (mode of delivery, gender, multiple gestation, first-attempt success, complication rate, airway trauma, and pneumothorax) are presented as counts or percentages and compared using the chi-square test or Fisher's exact test where expected cell counts were <5.

Multivariable analyses were performed using logistic regression, adjusting for clinically relevant covariates such as gestational age, gender, multiple gestation, antenatal steroids, delivery mode, 1-min Apgar score, and advanced airway group.

A two-sided *p*-value <0.05 was considered statistically significant. No formal adjustment for multiple comparisons was applied for secondary outcomes; these analyses are considered exploratory. Effect sizes are reported as mean differences for continuous variables, odds ratios (ORs) for categorical outcomes, and corresponding *p*-values or 95% confidence intervals (CIs).

A preplanned subgroup analysis was conducted for infants born <34 weeks’ gestation.

All analyses were conducted using standard statistical software (MedCalc Statistical Software 23.6.2, MedCalc Software Ltd., Ostend, Belgium)

## Results

### Study population

A total of 153 infants were enrolled; 73 in the SAD group and 80 infants in the ETT group. Baseline characteristics were similar between the two groups (Table 1). There were no statistically significant differences in gestational age (32.7 ± 2.3 vs. 32.0 ± 2.8 weeks, *p* = 0.13), birth weight (1.68 ± 0.37 vs. 1.63 ± 0.44 kg, *p* = 0.48), or 1-min Apgar score (median = 3, IQR = 1 for both groups, *p* = 0.83). The distribution of delivery mode and gender did not differ significantly between the groups. There was a statistically significant (*p* = 0.02) difference in multiple gestations between the groups.

**Table 1:** Baseline characteristics of infants by the airway method.

Characteristic	SAD (*n* = 73)	ETT (*n* = 80)	*p*-value
Gestational age (weeks), mean ± SD	32.0 ± 2.8	32.7 ± 2.3	0.13
Birth weight (kg), mean ± SD	1.63 ± 0.44	1.68 ± 0.37	0.48
Multiple gestation, %	15%^a^	31%	0.02
C/S delivery, %	79%	88%	0.19
Male sex, %	59%	59%	0.99
1-min Apgar score, median (IQR)	3 (1)	3 (1)	0.83

a Significant difference between the two groups, *P* < 0.05.

### Procedural variables

First-attempt success was higher in the SAD group than in the ETT group (99% vs. 90%), with a trend toward statistical significance (*p* = 0.055). As a result, time to successful airway placement was shorter with the SAD (9 ± 2 vs. 11 ± 3 s, *p* = 0.001) (Table 2).

**Table 2:** Procedural outcomes by the airway method.

Outcome	SAD (*n* = 73)	ETT (*n* = 80)	*p*-value
First-attempt success, %	99^a^	90	0.055
Number of attempts, mean ± SD	1.0 ± 0.1	1.1 ± 0.3	0.07
Time to successful placement (s)	9 ± 2^a^	11 ± 3	0.001
Time to return of spontaneous respiration (s)	45 ± 12	42 ± 8	0.07
5-min Apgar score, median (IQR)	6 (1)	5 (1)	0.493
10-min Apgar score, median (IQR)	9 (1)	8.5 (1)	0.665
Infants with procedural complications, %	18	23	0.55
Airway trauma, %	0%^a^	15%	0.004
Pneumothorax, %	3%	5%	0.68

a Significant difference between the two groups, *P* < 0.05.

### Clinical outcomes

The Apgar score at 5 and 10 min did not differ significantly between the groups. Time to return of spontaneous respiration was comparable between the groups (45 ± 12 s for the SAD vs. 42 ± 8 s for the ETT; *p* = 0.07). The overall rate of complications associated with the airway placement procedure was similar between the groups (18% vs. 23%, *p* = 0.55), but the rate of airway trauma was significantly different (0% vs. 15%, *p* = 0.004). The incidence of pneumothorax was similar between the groups (Table 2).

### Multivariable logistic regression

In adjusted analysis, use of an SAD was associated with increased odds of first-attempt success compared with the ETT, although this did not reach conventional statistical significance [adjusted odds ratio (aOR) 9.28, 95% CI 0.89–96.76, *p* = 0.063]. Cesarean delivery was independently associated with higher odds of first-attempt success (aOR 8.88, 95% CI 1.11–71.04, *p* = 0.040). Gestational age, gender, multiple gestation, antenatal steroids, delivery mode, and 1-min Apgar score were not independently associated with first-attempt success. The overall model demonstrated an acceptable fit (likelihood ratio *p* = 0.040) with moderate explanatory power (pseudo R^2^ = 0.22). Interaction terms (e.g., GA × Airway group) were not evaluated because of the low number of outcome events (Table 3).

**Table 3:** Multivariable logistic regression for first-time success.

Variable	aOR	95% CI	*p*-value
SAD (vs. ETT)	9.28	0.89–96.76	0.063
Cesarean delivery	8.88	1.11–71.04	0.040
1-min Apgar	0.86	0.39–1.91	0.718
Gestational age	0.82	0.48–1.42	0.486
Multiple gestation	0.42	0.06–2.72	0.362
Antenatal steroid	0.41	0.03–5.20	0.495
Male gender	0.32	0.05–1.91	0.211

### Subgroup analyses

Eighty-three infants were less than 34 weeks’ gestation (SAD = 47, ETT = 36) and comparisons were similar to the above (Table 4).

**Table 4:** Comparison of <34 weeks’ gestation subgroup.

Characteristic or Outcome	SAD (*n* = 47)	ETT (*n* = 36)	*p*-value
Gestational age (week), mean ± SD	30.7 ± 2.6	30.8 ± 2.3	0.86
Birth weight (kg), mean ± SD	1.44 ± 0.42	1.45 ± 0.41	0.92
First-attempt success, %	100	94	0.19
Number of attempts, mean ± SD	1.0 ± 0.0^a^	1.1 ± 0.2	0.001
Time to successful placement (s)	9 ± 2^a^	11 ± 4	0.004
Time to return of spontaneous respiration (s)	49 ± 13^a^	43 ± 9	0.02
10-min Apgar score, median (IQR)	9 (1)	8 (1)	0.28
Infants with procedural complications, %	23	28	0.80

a Significant difference between the two groups, *P* < 0.05.

## Discussion

In this cohort study of neonates requiring advanced airway support when they did not initially respond to FMV, the AerVia SAD demonstrated comparable overall effectiveness to the ETT, while offering clinically meaningful advantages in speed, first-attempt success rate, and safety profile. These advantages were also noted among infants born <34 weeks’ gestation. Placement of the AerVia SAD was significantly faster and required fewer attempts than ETT placement. Our findings are consistent with prior trials and systematic reviews comparing SADs with FMV or the ETT in late-preterm and term populations (2, 9, 16–18). The 2025 AHA/ILCOR guidelines already recognize SADs as a reasonable alternative to the ETT in infants ≥34 weeks when FMV fails (10, 11). Our data extend this finding by showing similar performance and safety in a group of preterm infants that includes many below 35 weeks’ gestation.

In adjusted multivariable logistic regression, SAD use was associated with substantially higher odds of first-attempt success compared with the ETT (aOR 9.28, 95% CI 0.89–96.76, *p* = 0.063), although this did not reach conventional statistical significance. Complication rates were comparable between the two groups, but the nature of complications differed markedly. Mild abdominal distension was the most common issue with AerVia. This is a known, usually self-limited consequence of SADs (19). Airway trauma was, however, more frequent with the ETT and consistent in earlier reports of laryngeal injury associated with intubation attempts in VLBW neonates (1, 5). These findings align with the growing body of evidence that SADs can reduce the technical burden and traumatic complications of advanced airway management without sacrificing efficacy (2, 8, 9, 17, 20, 21).

The potential clinical impact of these results is substantial, as SADs offer meaningful advantages for neonatal resuscitation across a wide range of clinical settings. These advantages would have particular importance in delivery scenarios where highly skilled neonatal resuscitators or advanced airway equipment may not be immediately available. This would include out-of-hospital deliveries potentially encountered by emergency medical technicians, institutions without neonatology services, and low- or middle-income countries. Pejovic et al. has demonstrated that SADs can be used safely and effectively by midwives during neonatal resuscitation in resource-limited settings (17). Even in high-resource settings, SADs provide meaningful benefits for nurses and other non-physician providers to deliver effective PPV when those skilled in laryngoscopy are not present at the bedside (22). By enabling rapid, high-success-rate resuscitation even by providers with limited advanced airway experience, the SAD has the potential to shorten critical response times, improve resuscitation efforts during the pivotal first minutes of life, and ultimately contribute to lives saved.

The importance of demonstrating comparable effectiveness in populations less than 34 weeks’ gestation is particularly important, given that this group experiences the highest burden of respiratory disease and faces the greatest risks from traditional laryngoscopy and endotracheal intubation (23, 24). In our <34-week cohort (mean gestational age 30.7 ± 2.6 weeks, mean birth weight 1.44 ± 0.42 kg), SAD placement achieved 100% first-attempt success. Although time to return of spontaneous respiration was statistically longer by approximately 6 s in the AerVia group (49 ± 13 vs. 43 ± 9 s), this small difference is unlikely to be clinically meaningful as 10-min Apgar scores were similar between the groups. These findings demonstrate the technical ease and feasibility of appropriately sized neonatal SADs even in preterm and very-low-birth-weight infants and directly address a key evidence gap for this high-risk population. To date, the effective use of supraglottic devices on smaller infants has not been one designed for this population (18, 25–32). The effective use of purpose-built devices designed for preterm infants has been demonstrated only in simulation (33). Ultimately, our data strengthen the case for expanded SAD application and thus the potential for improved resuscitation outcomes.

This study has several strengths. All data were collected prospectively. The study was conducted in a high-volume delivery center in a middle-income country with advanced neonatal services. Provider-specific procedural numbers were not prospectively recorded for each individual airway attempt. The high first-attempt ETT success rate observed in our cohort (90%) likely reflects the large volume of infants cared for and the extensive airway experience that providers have in our setting. We acknowledge that these findings may not be generalizable to all delivery settings, particularly those with limited access to experienced neonatal intubation providers. We have, however, provided pragmatic, real-world data from a setting where advanced airway skills may be variably available. Inclusion of infants across a wide gestational age range (including many <34 weeks) directly addresses the knowledge gap highlighted in current international guidelines (10, 11). The AerVia device itself represents a next-generation neonatal-specific SAD designed for the smallest patients and overcomes the size limitations of earlier laryngeal masks.

The limitations of our study include the non-randomized, single-center design and the use of a historical ETT comparison cohort. The historical ETT cohort was selected because this study represented our initial clinical implementation of a supraglottic airway device, and a contemporaneous randomized control group was not feasible during the device implementation phase. Both cohorts were managed within the same NICU by the same neonatal team using the same eligibility criteria, NRP-based delivery-room protocols, respiratory management strategies, surfactant administration practices, and outcome definitions. During the study period, there were no major changes in NICU organization, delivery-room protocols, respiratory support strategies, or staffing structure that would be expected to systematically influence outcomes between groups. However, we acknowledge that the AerVia cohort was evaluated after the historical ETT cohort, and increased provider familiarity, procedural experience, and team coordination over time may have introduced temporal bias.

We also acknowledge that device selection was not randomized, and no predefined criteria were established for choosing AerVia over the ETT. The choice of airway device was determined by the resuscitation team based on real-time clinical assessment. Therefore, differences in baseline severity between the two groups cannot be excluded and may have influenced the observed outcomes. All procedures were performed by neonatal providers experienced in neonatal resuscitation and airway management. Differences in case selection, clinical severity, and operator experience may have also contributed to outcome differences. Finally, both the overall sample size and the number of infants born at <34 weeks’ gestation were relatively modest. This limits the statistical power to detect small differences in resuscitation success or rare adverse events within this highest-risk group.

Taken together, however, these results suggest that appropriately sized neonatal-specific SADs may offer a feasible alternative to an ETT as a primary advanced airway option in neonatal resuscitation for infants <34 weeks’ gestation. Routine availability of such devices could meaningfully improve resuscitation efforts, particularly in settings where advanced airway expertise varies. However, given that several key outcomes did not reach statistical significance and the limitations inherent to the study design, larger multicenter randomized controlled trials are needed to confirm these findings and to better define the role of SADs in infants born <34 weeks’ gestation.

## Data Availability

The raw data supporting the conclusions of this article will be made available by the authors, without undue reservation.
